# Oxygenated Cembranoids from the Soft Coral *Sinularia flexibilis*

**DOI:** 10.3390/ijms14024317

**Published:** 2013-02-21

**Authors:** Ching-Chyuan Su, Bing-Sang Wong, Chuen Chin, Yu-Jen Wu, Jui-Hsin Su

**Affiliations:** 1 Department of Thoracic Cardiovascular Surgery, Antai Medical Care Cooperation, Antai Tian-Sheng Memorial Hospital, Pingtung 92842, Taiwan; E-Mail: a081001@mail.tsmh.org.tw; 2 Department of Deputy Superintendent, Antai Medical Care Cooperation, Antai Tian-Sheng Memorial Hospital, Pingtung 92842, Taiwan; E-Mails: a098123@mail.tsmh.org.tw (B.-S.W.); a096001@mail.tsmh.org.tw (C.C.); 3 Department of Beauty Science, Meiho University, Pingtung 91202, Taiwan; 4 National Museum of Marine Biology & Aquarium, Pingtung 94450, Taiwan; 5 Graduate Institute of Marine Biotechnology, National Dong Hwa University, Pingtung 94450, Taiwan

**Keywords:** diterpenoid, soft coral, *Sinularia flexibilis*

## Abstract

Chemical examination of the Taiwanese soft coral *Sinularia flexibilis* led to the isolation of five cembrane-based diterpenoids **1**–**5**, including two new metabolites, 11-acetylsinuflexolide (**1**) and 11-acetyldihydrosinuflexolide (**2**). The structures of the new metabolites were determined based on extensive spectroscopic analysis, particularly mass spectrometry and 2D NMR (^1^H–^1^H COSY, HMQC, HMBC, and NOESY) spectroscopy. Metabolites **1**, **3** and **4** exhibited moderate to weak cytotoxicity to human tumor cell lines, HeLa, HEp-2, MCF-7 and MDA-MB-231.

## 1. Introduction

**Figure 1:**
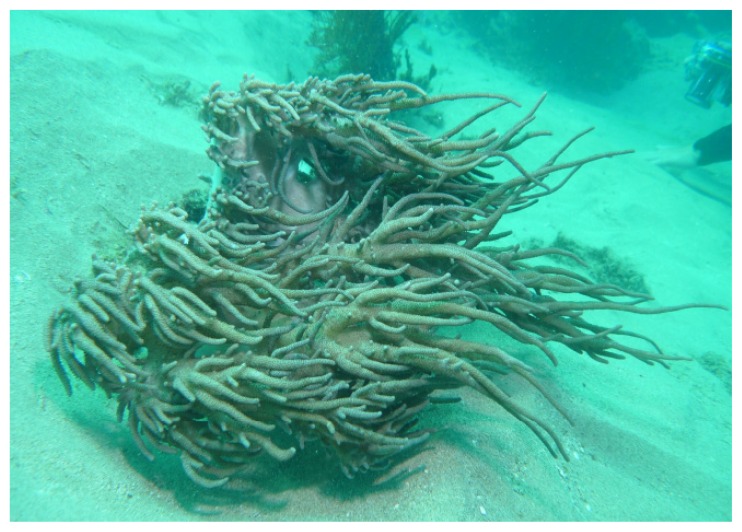
The soft coral *Sinularia flexibilis.*

**Figure 2:**
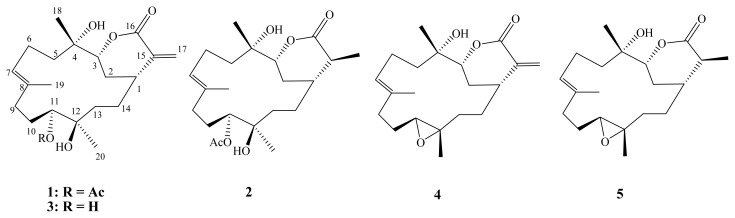
Structures of metabolites **1**–**5**.

Soft corals have attracted a great deal of attention in light of the structural diversity and wide range of biological activities of their metabolites [1]. Recently, in the investigation of the bioactive metabolites from the Formosan soft corals, many bioactive cembranoids have been isolated from soft corals (Alcyonaceae) belonging to the genera *Sinularia* [2–11], *Sarcophyton* [12–16] and *Lobophytum* [17–19]. Some of these metabolites have been found to possess several kinds of biological activities, such as cytotoxic [4,9–18] and anti-inflammatory activity [2–8,12–14,18,19]. During the course of our investigation on new natural substances from wild and cultured soft coral *Sinularia flexibilis*, a number of cembrane-based diterpenoids were discovered, and some were found to be bioactive [20]. In continuation of our search for biologically active secondary metabolites from a soft coral *Sinularia flexibilis* (Figure 1), we have isolated two new cembrane-based diterpenoids, 11-acetylsinuflexolide (**1**) and 11-acetyldihydrosinuflexolide (**2**), along with three known cembranoids, sinuflexolide (**3**) [21], sinularin (**4**) [22] and dihydrosinularin (**5**) [22] (Figure 2). The structures of **1** and **2** were established by extensive spectroscopic analysis, including careful examination of 2D-NMR (^1^H–^1^H COSY, HMQC, HMBC and NOESY) (Figures S1–S10) correlations and by comparison of their NMR data with those of related compounds. The cytotoxicity of compounds **1**–**5** against human cervical epitheloid carcinoma (HeLa), laryngeal carcinoma (HEp-2) and breast carcinoma (MCF-7 and MDA-MB-231) cell lines was also investigated.

## 2. Results and Discussion

Frozen samples of *Sinularia flexibilis* were extracted with EtOAc. The dry EtOAc extracts were fractionated by silica gel gravity column chromatography, and the eluted fractions were further purified by HPLC to yield cembranoids **1**–**5**.

**Figure 3:**
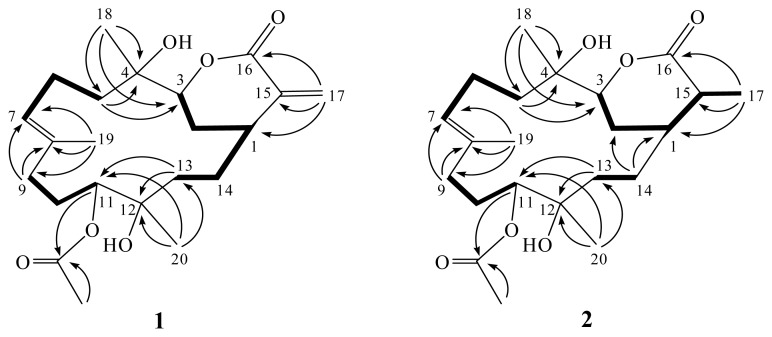
The structures of metabolites **1** and **2** and selected ^1^H-^1^H COSY (−) and HMBC (→) correlations.

The HR-ESI-MS (*m*/*z* 417.2250 [M + Na]^+^) of 11-acetylsinuflexolide (**1**) established the molecular formula C_22_H_34_O_6_, appropriate for six degrees of unsaturation. Inspection of the ^13^C-NMR and DEPT spectroscopic data (Table 1) (Figures S1 and S2) showed signals of four methyls (including one acetate methyl), seven sp^3^ methylenes, one sp^2^ methylene, three sp^3^ methines (including two oxymethines), one sp^2^ methine, two sp^3^ and four sp^2^ quaternary carbons (including two ester carbonyls). The ^13^C NMR signals appearing at δ_C_ 166.6 (C), 140.4 (C), 125.5 (CH_2_), 84.5 (CH), 36.7 (CH), and 29.3 (CH_2_) were assigned to an α-exomethylenic–δ-lactone ring functionality by comparing the very similar NMR data of the cembranoids with the same six-membered lactone ring [23,24]. Resonances in the ^13^C NMR spectrum of **1** at δ_C_ 170.6 (C) supported the presence of one additional ester group (Table 1). The ester was identified as acetate by the presence of one methyl resonance in the ^1^H NMR spectrum at δ_H_ 2.11 (3H, s) (Table 1). Furthermore, carbon signals of three methyls (δ_C_ 16.1, 25.4 and 25.5), one trisubstituted double bond (δ_C_ 135.1, C; 127.2, CH), two oxygen-bearing methines (δ_C_ 84.5 and 77.5), and two oxygenated quaternary carbons (δ_C_ 74.8 and 73.7) were also determined. The ^1^H-NMR spectral data revealed the presence of two olefinic methylene protons (δ 6.43, d, *J* = 2.0 Hz and 5.63, d, *J* = 2.0 Hz) and one olefinic methine proton (δ 5.26, dd, *J* = 7.5, 7.5 Hz). Furthermore, two oxygenated methines (δ 4.79, dd, *J* = 6.5, 2.5 Hz and 4.05, d, *J* = 11.5 Hz) were also designated from the ^1^H NMR signals. By interpretation of ^1^H-^1^H COSY correlations (Figure S5), it was possible to establish three partial structures from H-1 to H-3, from H_2_-5 to H-7, from H_2_-9 to H-11, and from H_2_-13 to H-1 through H_2_-14 (Figure 3). These data, together with the HMBC correlations (Figure 3) (Figure S4) from H_2_-5 to C-3 and C-4, H_2_-9 to C-7 and C-8, and H_2_-13 to C-11 and C-12 established the connectivity within the 14-membered ring. Three methyl groups attached at C-4, C-8 and C-12 were confirmed by the HMBC correlations from H_3_-18 to C-3, C-4 and C-5, H_3_-19 to C-7, C-8 and C-9, H_3_-20 to C-11, C-12 and C-13. A 1,1-disubstituted double bond attached at C-15 was confirmed by the HMBC correlations from H_2_-17 to C-1, C-15 and C-16. Moreover, one acetoxy group positioned at C-11 was confirmed from the HMBC correlations of H-11 (δ 4.79) and protons of an acetate methyl (δ 2.11) to the ester carbonyl carbon at δ 170.6 (C). The *E*–configuration of one double bond at C-7/C-8 was assigned based on the ^13^C NMR chemical shifts at C-19 (δ_C_ 16.1). Thus, **1** was revealed as a cembranoid possessing an α-exomethylenic–δ-lactone ring, based on the above analysis. Furthermore, the relative stereochemistry of **1** was mostly confirmed to be the same as that of the known metabolite sinuflexolide (**3**) by comparison of the proton chemical shifts and coupling constants [24]. Further comparison of the ^1^H and ^13^C NMR data of **1** with those of **3**, showed that **1** contains an extra acetyl group relative to **3**. The chemical shift of H-11 in **3** (δ_H_ 3.47, dd, *J* = 6.4, 2.4 Hz) was shifted downfield (δ_H_ 4.79, dd, *J* = 6.5, 2.5 Hz) in **1**, suggesting that **1** is the 11-acetyl derivative of **3**. This was further supported by acetylation of **3** with acetic anhydride in pyridine to yield **1**. Thus, compound **1** was established as the 11-acetyl derivative of **3**.

11-acetyldihydrosinuflexolide (**2)** obtained as a white powder. The HRESIMS (*m*/*z* 419.2411, [M + Na]^+^) and NMR data of **2** indicated the molecular formula, C_22_H_36_O_6_. Both the ^1^H and ^13^C NMR signals of **2** were found to be very closely related to those of compound **1**, suggesting the same skeleton. Further comparison of NMR data of **2** with those of **1** (Table 1) (Figures S1–S10), revealed that the two exomethylene proton signals (δ_H_ 6.43 and 5.63) in **1** was replaced by a methyl proton signal (δ_H_ 1.35 d, *J* = 7.0 Hz) in **2**. This was confirmed by the HMBC correlations (Figure 3) from H_3_-17 to C-1, C-15 and C-16. The relative stereochemistry of all stereocenters except C-15 of **2** was determined to be the same as that of **1** by comparison of the proton shifts and coupling constants. The methyl group at C-15 was assigned the β-configuration primarily due to the NOE correlation between H_3_-17 and H-1. Furthermore, comparison of the NMR data between **2** and **5** confirmed both compounds have the same relative stereochemistry at C-15 [22]. Thus, the structure of **2** was established.

Finally, a 3-(4,5-dimethylthiazol-2-yl)-2,5-diphenyl tetrazolium bromide (MTT) assay was used to examine the cytotoxic activities of compounds 1–5 against four cancer cell lines, including human cervical epitheloid carcinoma (HeLa), laryngeal carcinoma (HEp-2) and breast carcinoma (MCF-7 and MDA-MB-231) cancer cells. Cells were treated with different concentrations of 1–5 for 72 h. The results show that compound 3, the most potent of compounds 1–5, exhibited cytotoxicity against the HeLa, HEp-2, MCF-7 and MDA-MB-231 cancer cell lines with IC50 values of 8.6, 8.2, 16.0 and 11.3 μg/mL, respectively. Furthermore, compounds 1 and 4 were found to exhibit weak cytotoxicity towards some of the cell lines (Table 2).

## 3. Experimental Section

### 3.1. General Procedures

Optical rotation values were measured using a Jasco P-1010 digital polarimeter. IR spectra were recorded on a Varian Digilab FTS 1000 Fourier transform infrared spectrophotometer. NMR spectra were recorded on a Varian Mercury Plus 400 FT-NMR (or Varian Unity INOVA 500 FT-NMR) instrument at 400 MHz (or 500 MHz) for ^1^H-NMR and 100 MHz (or 125 MHz) for ^13^C-NMR, respectively, in CDCl_3_. ESIMS and HRESIMS data were recorded with a Bruker APEX II mass spectrometer. Gravity column chomatography was performed on silica gel (230–400 mesh, Merck, Darmstadt, Germany). Thin layer chomatography (TLC) was carried out on precoated Kieselgel 60 F254 (0.2 mm, Merck) and spots were visualized by spraying with 10% H_2_SO_4_ solution followed by heating. HPLC was performed using a system comprised of a Hitachi L-7100 pump (Tokyo, Japan) and a Rheodyne 7725 injection (Cotati, CA, USA) port. A preparative normal phase column (Hibar 250 × 21.2 mm, Supelco, silica gel 60, 5 μm, Bellefonte, PA, USA) was used for HPLC.

### 3.2. Animal Material

The marine soft coral *Sinularia flexibilis* (Quoy and Gaimard, 1833) was collected by scuba divers at a depth of around 10 m off the coast of Pingtung County, Taiwan, in July 2012, and the sample was frozen immediately after collection. A voucher sample was deposited at the National Museum of Marine Biology and Aquarium, Taiwan (specimen No. 2012-0709-10).

### 3.3. Extraction and Separation

The soft coral (2.0 kg, wet wt.) was stored frozen and then freeze dried. The freeze-dried material (450 g) was minced and extracted five times with EtOAc (2 L) for 24 h each time at room temperature. The organic extract was evaporated to yield a residue (60.5 g), which was subjected to open column chomatography on silica gel eluting with gradients of *n*-hexane (H)–EtOAc (E), to give 14 fractions: Fr-1 (eluted by *n*-hexane), Fr-2 (eluted by H–E 100:1), Fr-3 (eluted by H–E 50:1), Fr-4 (eluted by H–E 30:1), Fr-5 (eluted by H–E 20:1), Fr-6 (eluted by H–E 15:1), Fr-7 (eluted by H–E 10:1), Fr-8 (eluted by H–E 8:1), Fr-9 (eluted by H–E 5:1), Fr-10 (eluted by H–E 3:1), Fr-11 (eluted by H–E 2:1), Fr-12 (eluted by H–E 1:1), Fr-13 (eluted by H–E 1:2), and Fr-14 (eluted by EtOAc). Fraction 10 was further separated by silica gel column chomatography with gradient elution (*n*-hexane–EtOAc, 5:1 to 1:1) to yield five subfractions (10A–E). Subfraction 10C was subjected to normal phase HPLC with *n*-hexane–EtOAc (4:1) as the eluent (flow rate 2 mL/min) to obtain compounds **4** (250 mg, 0.41% dry wt. of extract) and **5** (330 mg, 0.55% dry wt. of extract). Fraction 12 was further separated by silica gel column chomatography with gradient elution (*n*-hexane–EtOAc, 1:1 to 1:3) to yield seven subfractions (12A–G). Subfraction 12C was subjected to normal phase HPLC with *n*-hexane–EtOAc (1:1) as the eluent (flow rate 2 mL/min) to obtain compounds **1** (8.0 mg, 0.013% dry wt. of extract) and **2** (6.5 mg, 0.011% dry wt. of extract). Subfraction 12F was subjected to normal phase HPLC with *n*-hexane–acetone (1:1) as the eluent (flow rate 2 mL/min) to obtain compound **3** (6.5 mg, 0.011% dry wt. of extract).

11-Acetylsinuflexolide (**1**): white solid; mp 82.0–85.0 °C; [*α*]_D_^25^ −12 (*c* 0.7, CHCl_3_); IR (neat) *ν*_max_ 3434, 2974, 2937, 1712, 1622, 1452, 1376 and 1256 cm^−1^; UV (MeOH) *λ*_max_ (log *ɛ*) 212 (3.9) nm; ^13^C and ^1^H NMR data, see Table 1; ESIMS *m*/*z* 417 [M + Na]^+^; HRESIMS *m*/*z* 417.2250 [M + Na]^+^ (calcd for C_22_H_34_O_6_Na, 417.2253).

11-Acetyldihydrosinuflexolide (**2**): white solid; mp 75.0–78.0 °C; [*α*]_D_^25^−15 (*c* 0.6, CHCl_3_); IR (neat) *ν*_max_ 3434, 2975, 2938, 1714, 1639, 1458, 1377 and 1245 cm^−1; 13^C and ^1^H NMR data, see Table 1; ESIMS *m*/*z* 419 [M + Na]^+^; HRESIMS *m*/*z* 419.2411 [M + Na]^+^ (calcd for C_22_H_36_O_6_Na, 419.2409).

Sinuflexolide (**3**): white solid; [*α*]_D_^24^ −7.0 (*c* 0.2, CHCl_3_); IR (neat) *ν*_max_ 3400, 2972, 1714, 1458, 1381, and 1235 cm^−1^; UV (MeOH) *−*_max_ 215 (log *ɛ* =3.8); [lit. [*α*]_D_^25^ −8.6 (*c* 0.17, CHCl_3_)] [21].

Sinularin (**4**): white solid; [*α*]_D_^25^ –105 (*c* 1.0, CHCl_3_); IR (neat) *ν*_max_ 3404, 2965, 1710, 1455, 1381, and 1237 cm^−1^; UV (MeOH) *λ*_max_ 212 (log *ɛ* = 3.8); [lit. [*α*]_D_^20^ −127] [22].

Dihydrosinularin (**5**): white solid; [*α*]_D_^25^ –30 (*c* 0.3, CHCl_3_); IR (neat) *ν*_max_ 3400, 2960, 1714, 1459, 1385, and 1231 cm^−1^; [lit. [*α*]_D_^20^ −45] [22].

Acetylation of **3**: A solution of **3** (5.0 mg) in pyridine (0.5 mL) was mixed with Ac_2_O (0.1 mL), and stirred at room temperature for 24 h. After evaporation of excess reagent, the residue was subjected to column chromatograph over silica gel using *n*-hexane–EtOAc (1:2) to give the acetyl derivative **1** (4.8 mg, 81%).

### 3.4. Cytotoxicity Testing

Cell lines were purchased from the American Type Culture Collection (ATCC). Cytotoxicity assays of compounds **1**–**5** were performed using the 3-(4,5-dimethylthiazol-2-yl)-2,5-diphenyltetrazolium bromide (MTT) colorimetric method [25,26].

## 4. Conclusions

In the previous reports, cembranoids possessing a δ-lactone have been mostly isolated from soft coral (Alcyonaceae) belonging to the genera *Sinularia* [2,21–23,27,28] and *Lobophytum* [29]. Some of these metabolites have been found to possess several kinds of biological activities, such as cytotoxic [21–23] and anti-inflammatory activity [2,29]. In the present study, compounds **1**, **3** and **4** exhibited moderate or weak cytotoxicity against the growth of HeLa, HEp-2, MCF-7 and MDA-MB-231 cancer cell lines. According to the structures of **1**–**5**, it seems that the α-exomethylenic–ä-lactone ring group in compounds **1**, **3** and **4** is critical for the cytotoxic activity of metabolites **1**–**5**.

## Figures and Tables

**Table 1:** ^1^H and ^13^C NMR data for **1** and **2**.

C/H	1		2	
	
	δ_H_ (*J* in Hz) a	δ_C_ (mult.) b	δ_H_ (*J* in Hz) a	δ_C_ (mult.) b
1	2.75 m	36.7 (CH)	1.71 m	38.4 (CH)
2	2.25 m; 1.57 m	29.3 (CH_2_)	2.24 m; 1.44 m	29.8 (CH_2_)
3	4.05 d (11.5)	84.5 (CH)	4.05 d (11.5, 2.5)	85.0 (CH)
4		73.7 (C)		73.7 (C)
5	1.83 m; 1.77 m	37.8 (CH_2_)	1.77 m	37.5 (CH_2_)
6	2.28 m; 2.02 m	22.1 (CH_2_)	2.28 m; 1.98 m	22.1 (CH_2_)
7	5.26 dd (7.5, 7.5)	127.2 (CH)	5.23 dd (7.0, 7.0)	127.2 (CH)
8		135.1 (C)		135.0 (C)
9	2.31 m; 1.93 m	35.3 (CH_2_)	2.27 m; 1.92 m	35.1 (CH_2_)
10	1.92 m; 1.72 m	27.9 (CH_2_)	1.90 m; 1.72 m	28.1 (CH_2_)
11	4.79 dd (6.5, 2.5)	77.5 (CH)	4.80 dd (7.0, 2.0)	77.2 (CH)
12		74.8 (C)		74.8 (C)
13	1.74 m; 1.53 m	35.2 (CH_2_)	1.68 m; 1.48 m	36.1 (CH_2_)
14	1.92 m; 1.36 m	28.6 (CH_2_)	1.68 m; 1.12 m	28.7 (CH_2_)
15		140.4 (C)	2.09 m	43.5 (CH)
16		166.6 (C)		174.8 (C)
17	6.43 d (2.0); 5.63 d (2.0)	125.5 (CH_2_)	1.35 d (7.0)	15.3 (CH_3_)
18	1.38 s	25.5 (CH_3_)	1.39 s	25.6 (CH_3_)
19	1.62 s	16.1 (CH_3_)	1.62 s	16.4 (CH_3_)
20	1.19 s	25.4 (CH_3_)	1.17 s	25.4 (CH_3_)
OAC		170.6 (C)		170.6 (C)
	2.11 s	21.1 (CH_3_)	2.11 s	21.1 (CH_3_)

a 500 MHz in CDCl_3_;

b 125 MHz in CDCl_3_.

**Table 2:** Cytotoxicity (IC_50_ μg/mL) of compounds **1**–**5**.

Compound	Cell Lines			

	HeLa	HEp-2	MCF-7	MDA-MB-231
**1**	9.5	11.3	17.8	15.7
**2**	NA b	NA b	NA b	NA b
**3**	8.6	8.2	16.0	11.3
**4**	NA b	12.6	17.5	13.5
**5**	NA b	NA b	NA b	NA b
**Doxorubicin**^a^	0.05	0.1	0.07	0.5

a Clinical anticancer drug as positive control;

b NA, not active at 20 μg/mL.

## Supplementary Files

Supplementary File 1 Supporting Information (PDF, 230 KB)
