# Supplementary material for: Oxygenated Cembranoids from the Soft Coral Sinularia flexibilis
Source: Int J Mol Sci. 2013 Feb 21;14(2):4317–25. doi: 10.3390/ijms14024317 (PMC3588100; doi:10.3390/ijms14024317)
Supplement: Supplementary File 1 — Supporting Information (PDF, 230 KB) [file ijms-14-04317-s001.pdf]

Supporting Information

Figure S1. ^{1}H NMR spectrum of **1** in $CDCl_3$ at 500 MHz.

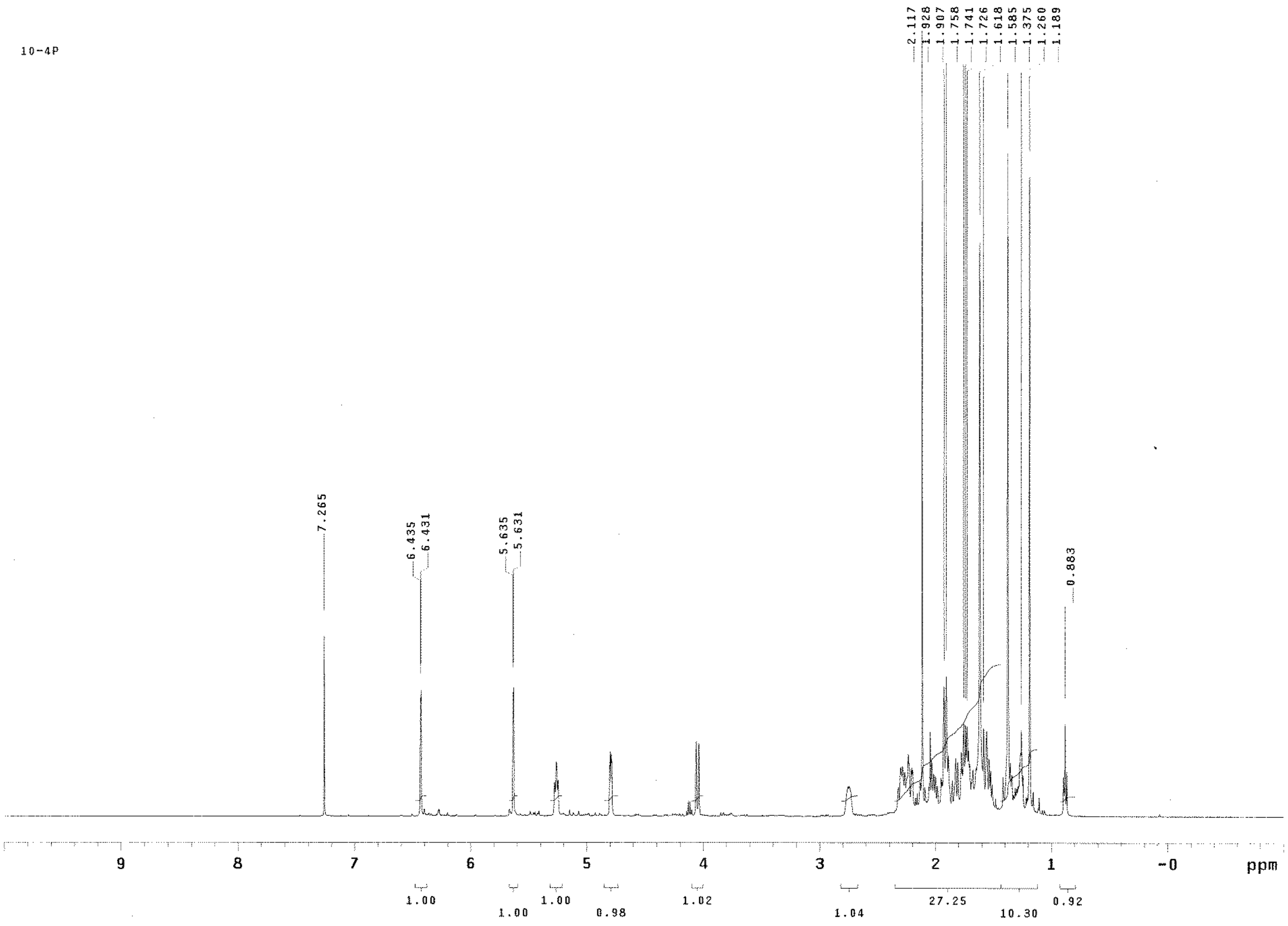

Figure S2. ^{13}C NMR spectrum of **1** in $CDCl_3$ at 125 MHz.

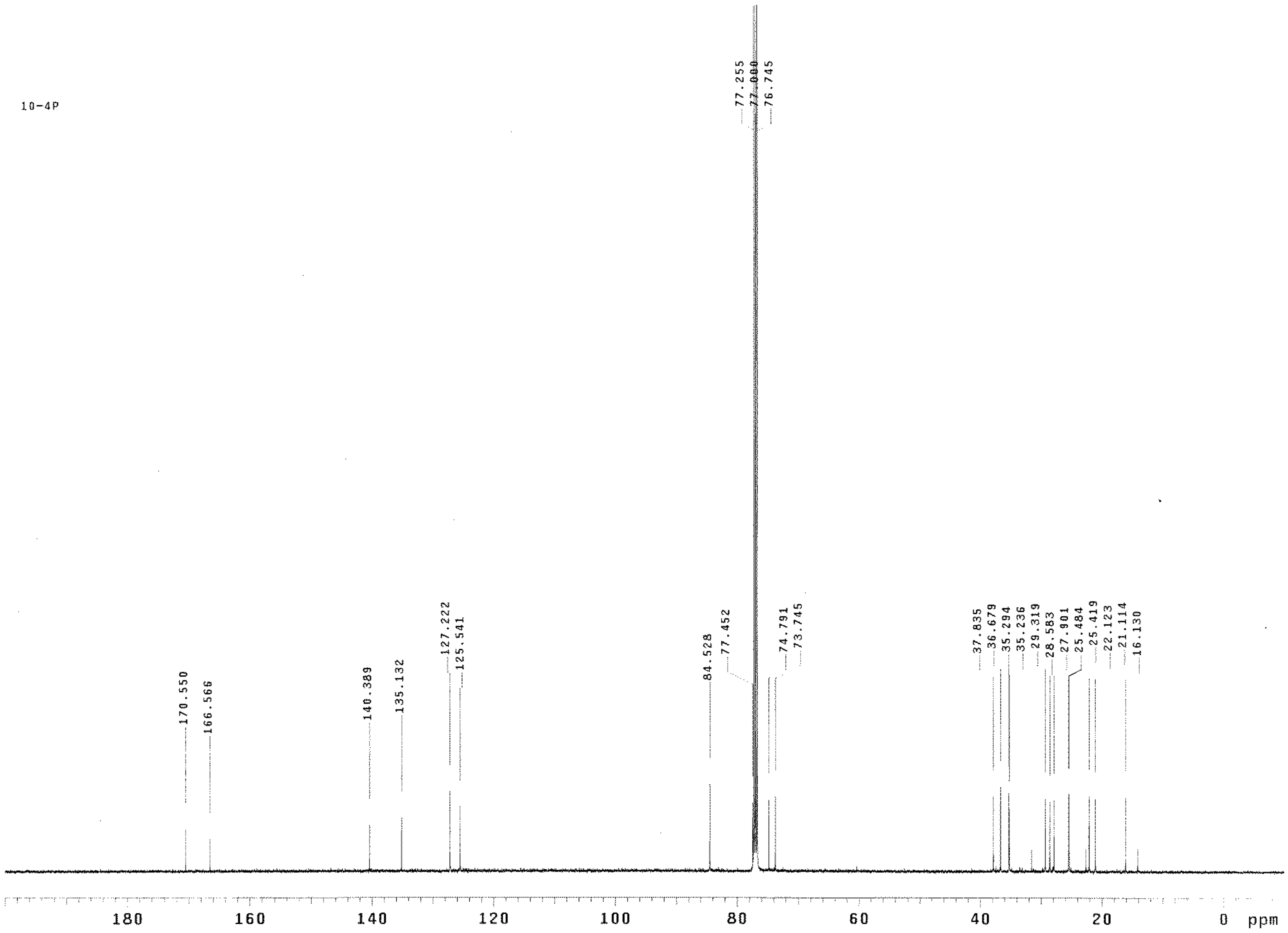

Figure S3. HMQC spectrum of **1** in $CDCl_3$.

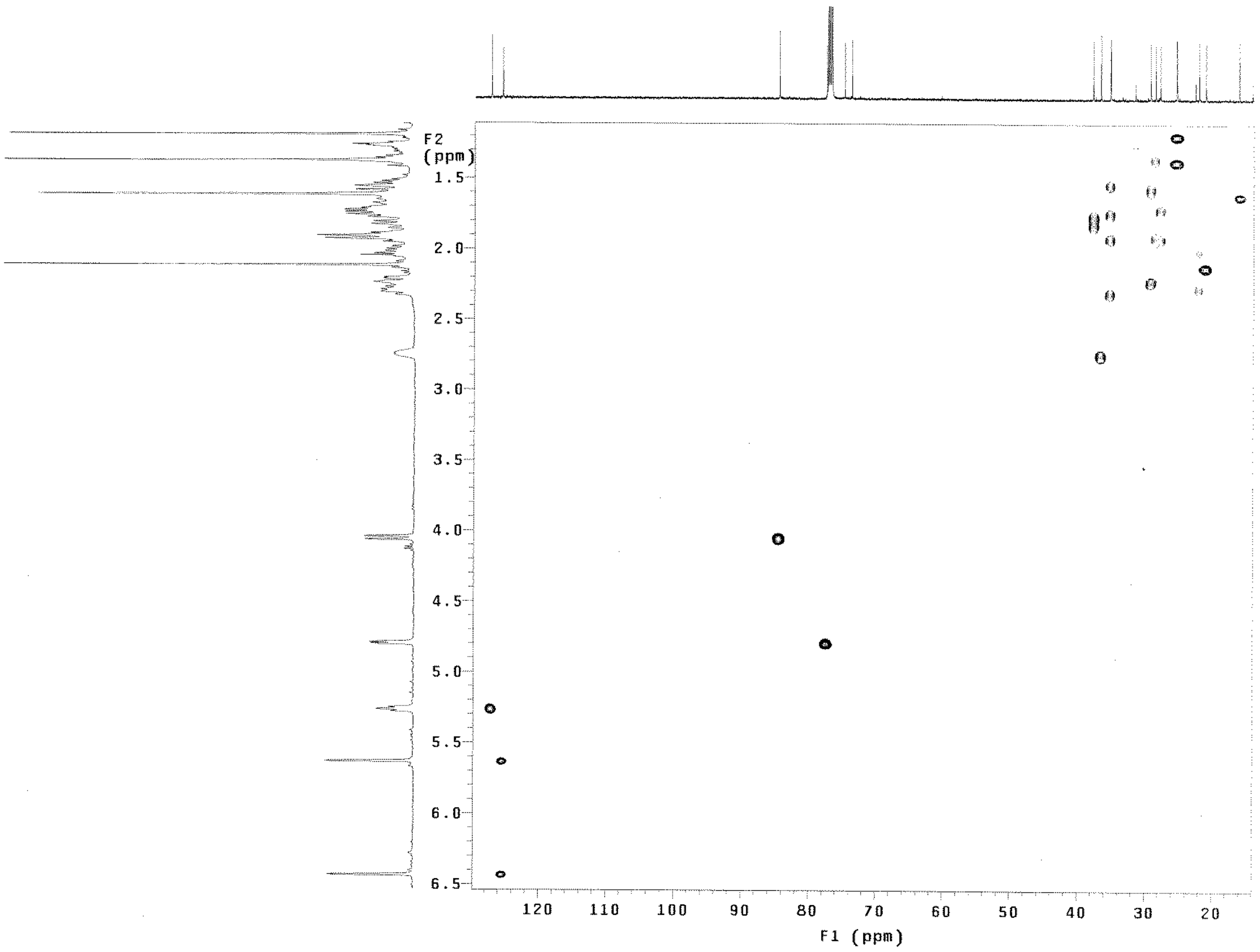

Figure S4. HMBC spectrum of **1** in $CDCl_3$.

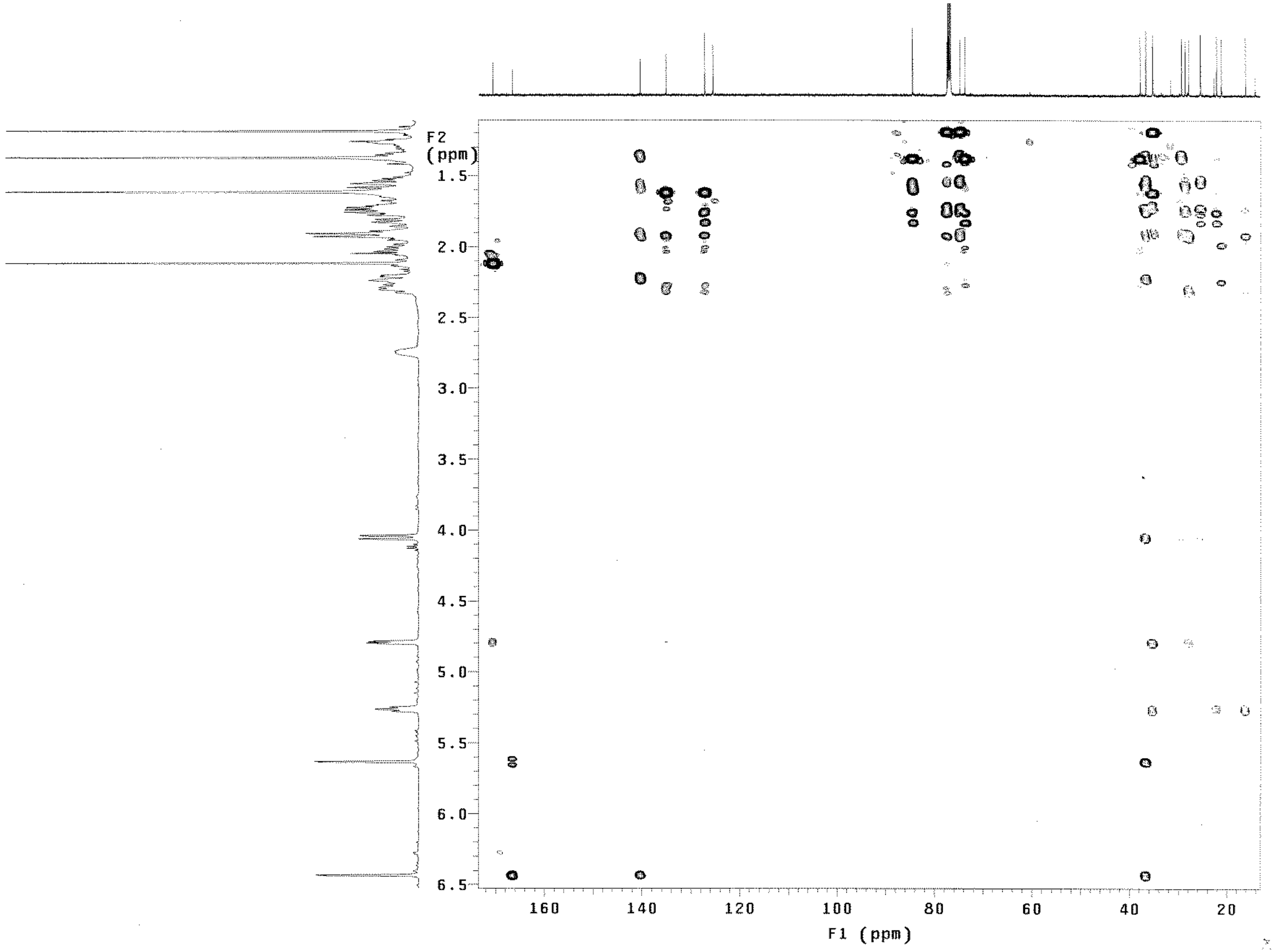

Figure S5. ^1H–^1H COSY spectrum of **1** in $CDCl_3$.

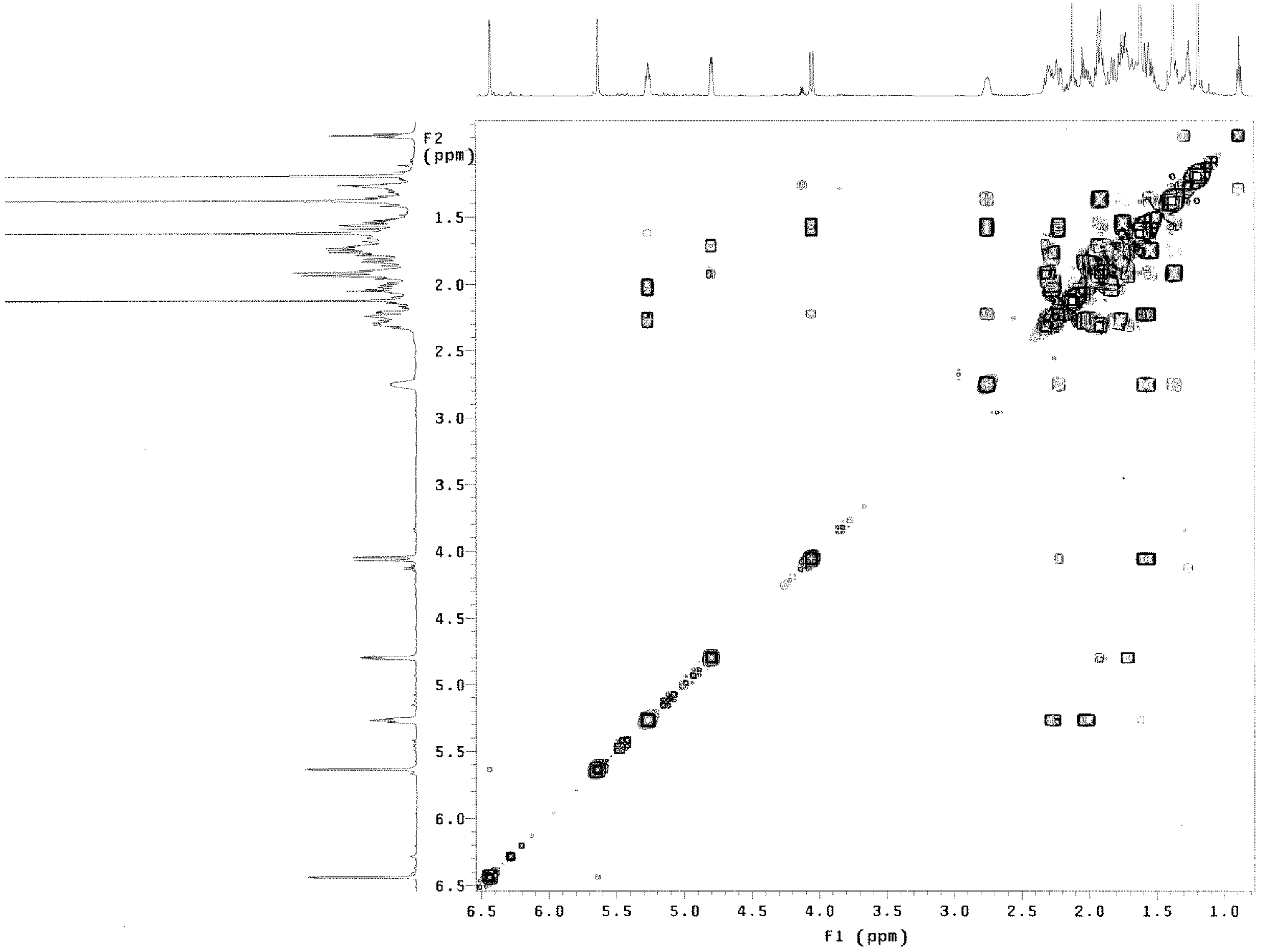

Figure S6. ^{1}H NMR spectrum of **2** in $CDCl_3$ at 500 MHz.

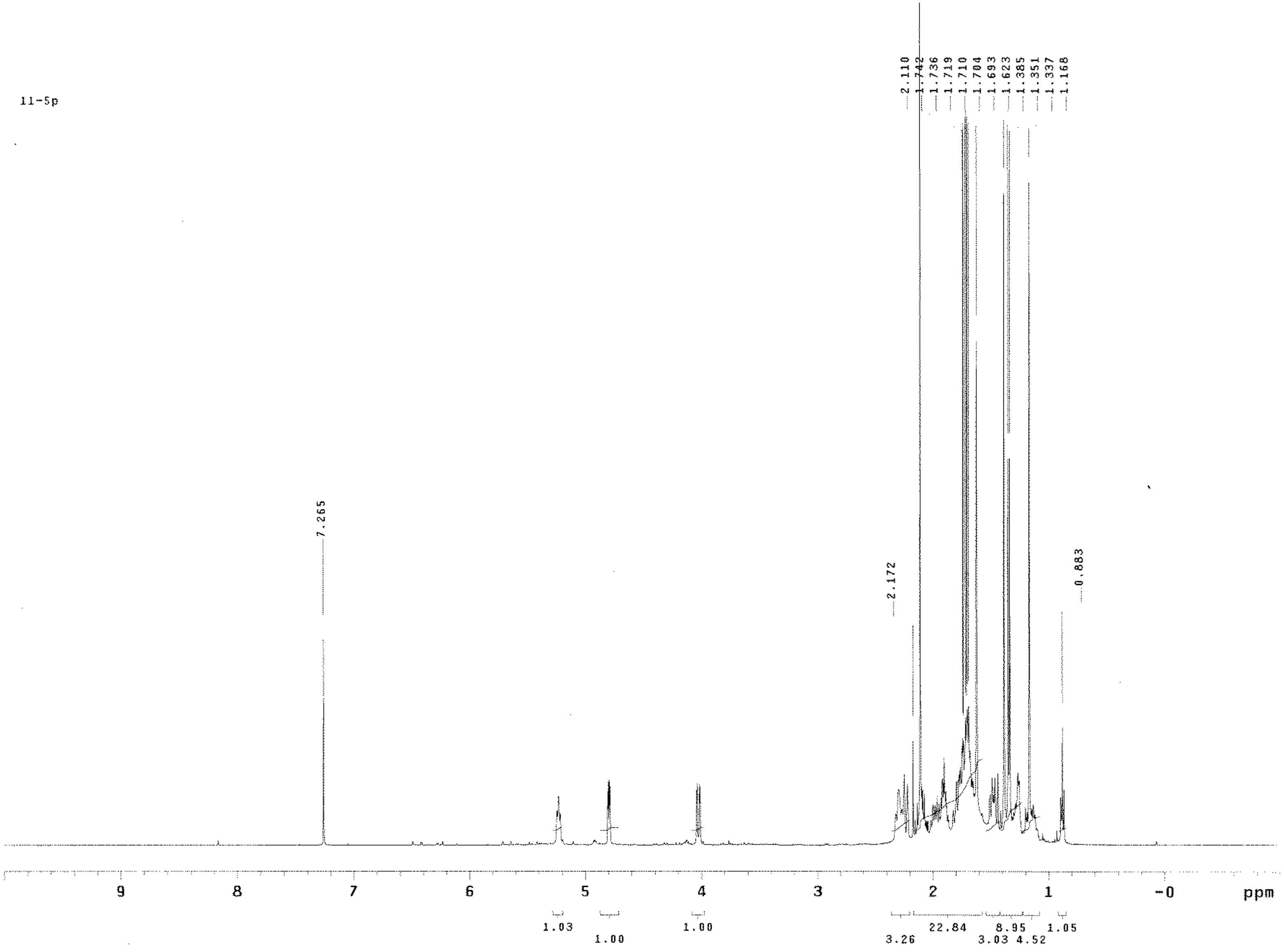

Figure S7. ^{13}C NMR spectrum of **2** in $CDCl_3$ at 125 MHz.

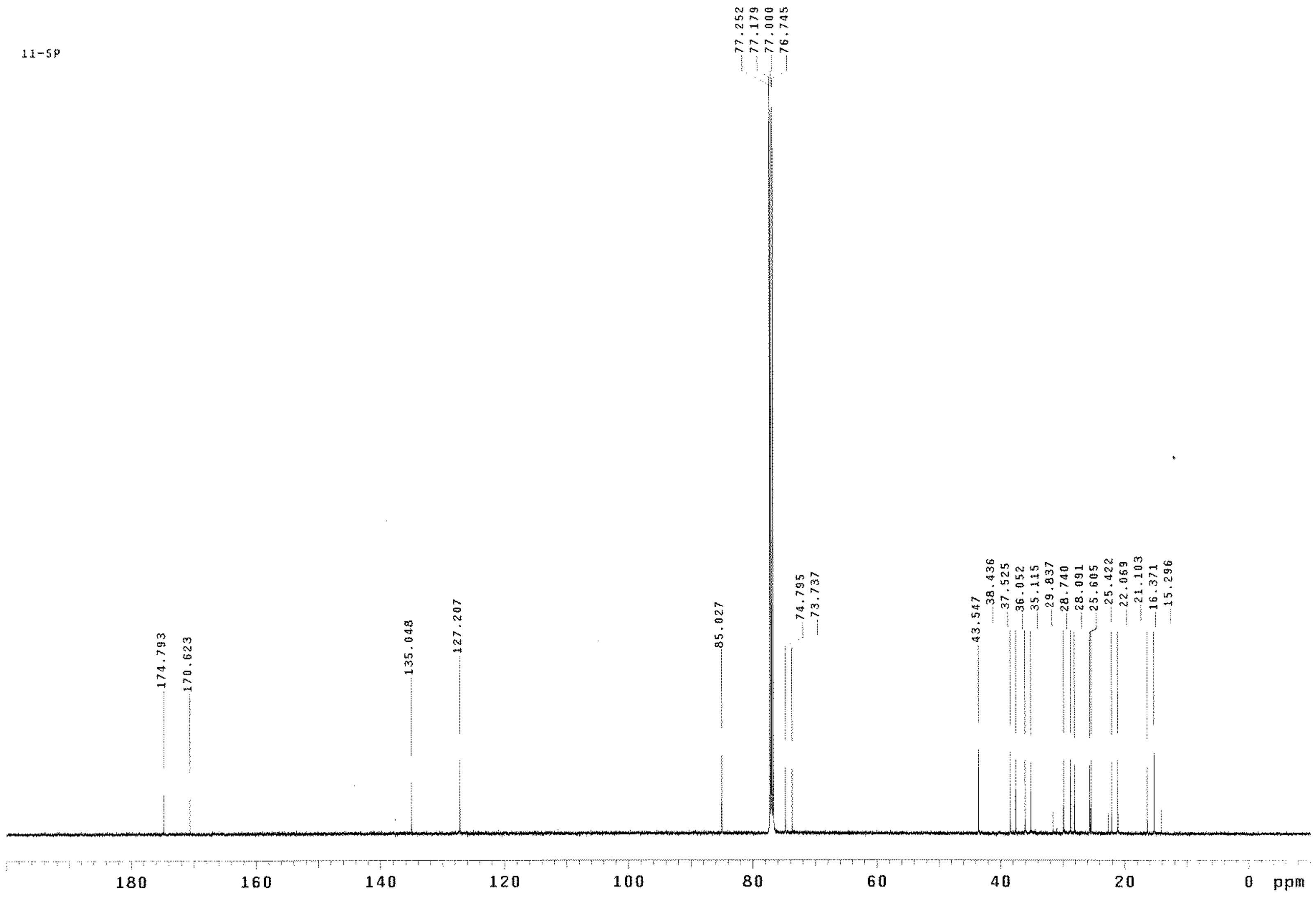

Figure S8. HMQC spectrum of **2** in $CDCl_3$.

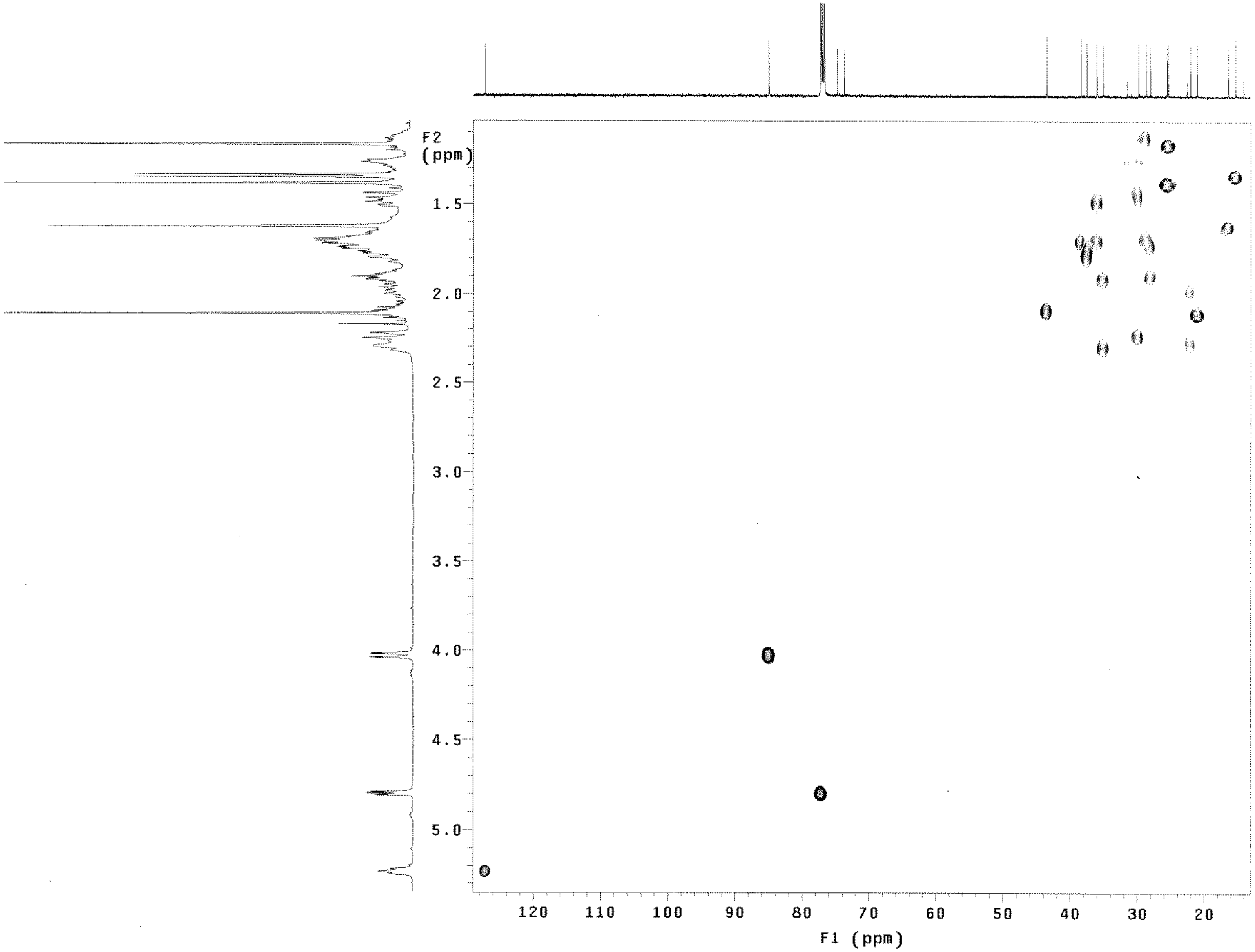

Figure S9. HMBC spectrum of **2** in $CDCl_3$.

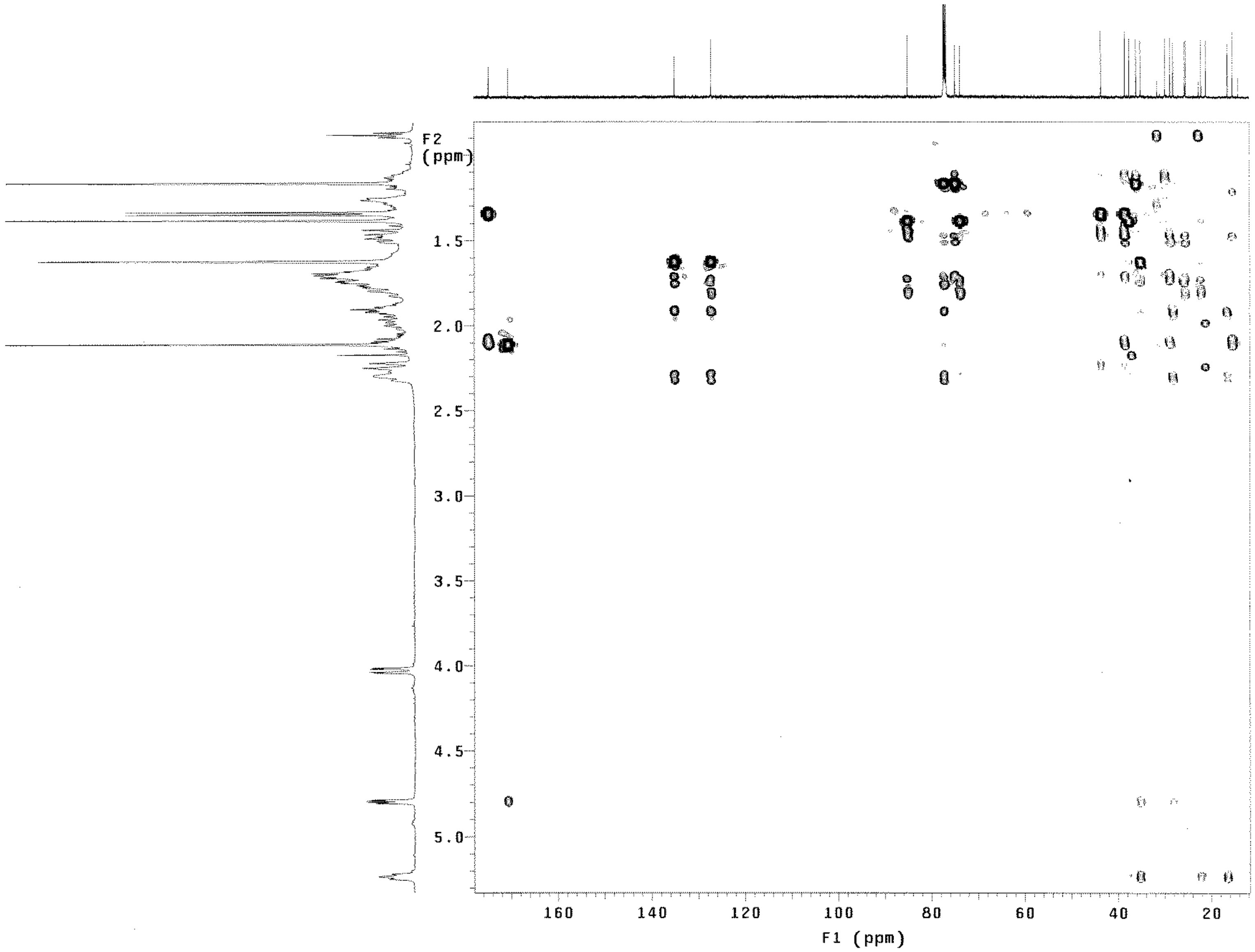

Figure S10. ^{1}H–^{1}H COSY spectrum of **2** in $CDCl_3$.

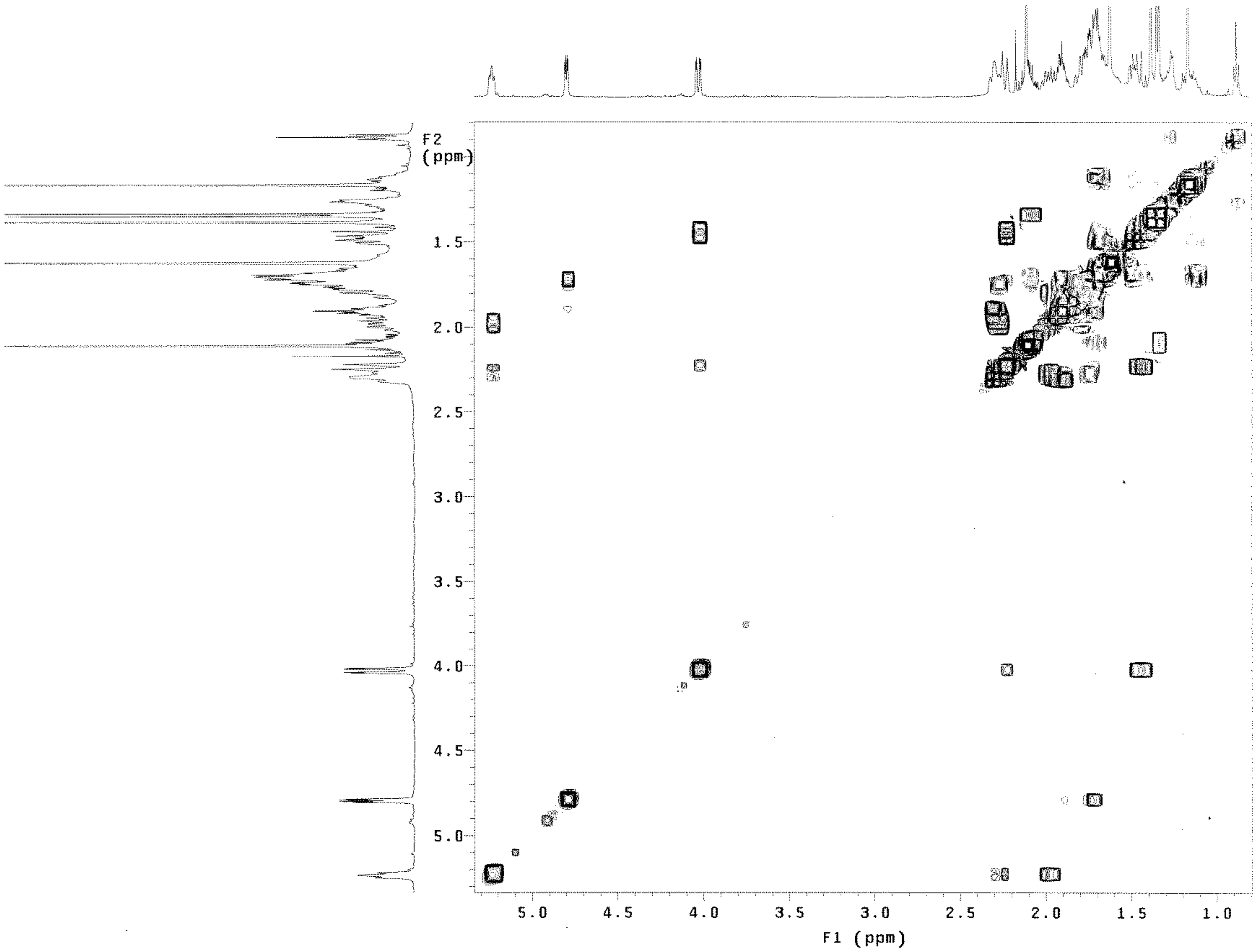

© 2013 by the authors; licensee MDPI, Basel, Switzerland. This article is an open access article distributed under the terms and conditions of the Creative Commons Attribution license (http://creativecommons.org/licenses/by/3.0/).
